# Intratumoural immunotherapies for unresectable and metastatic melanoma: current status and future perspectives

**DOI:** 10.1038/s41416-020-0994-4

**Published:** 2020-07-27

**Authors:** Mark R. Middleton, Christoph Hoeller, Olivier Michielin, Caroline Robert, Caroline Caramella, Katarina Öhrling, Axel Hauschild

**Affiliations:** 1 University of Oxford Department of Oncology, Old Road Campus Research Building, Roosevelt Drive, Oxford, UK; 2 Department of Dermatology, Medical University of Vienna, Vienna, Austria; 3 Department of Oncology, Lausanne University Hospital, Lausanne, Switzerland; 4 Department of Oncology, Gustave Roussy Cancer Campus, Villejuif, and Paris-Saclay University, Orsay, France; 5 Department of Radiology, Gustave Roussy Cancer Campus, Villejuif, France; 6 Amgen Europe GmbH, Rotkreuz, Switzerland; 7 Department of Dermatology, Venereology and Allergology, University Hospital Schleswig-Holstein, Kiel, Germany

**Keywords:** Melanoma, Metastasis

## Abstract

The emergence of human intratumoural immunotherapy (HIT-IT) is a major step forward in the management of unresectable melanoma. The direct injection of treatments into melanoma lesions can cause cell lysis and induce a local immune response, and might be associated with a systemic immune response. Directly injecting immunotherapies into tumours achieves a high local concentration of immunostimulatory agent while minimising systemic exposure and, as such, HIT-IT agents are associated with lower toxicity than systemic immune checkpoint inhibitors (CPIs), enabling their potential use in combination with other therapies. Consequently, multiple HIT-IT agents, including oncolytic viruses, pattern-recognition receptor agonists, injected CPIs, cytokines and immune glycolipids, are under investigation. This review considers the current clinical development status of HIT-IT agents as monotherapy and in combination with systemic CPIs, and the practical aspects of administering and assessing the response to these agents. The future of HIT-IT probably lies in its use in combination with systemic CPIs; data from Phase 2 trials indicate a synergy between HIT-IT and CPIs. Data also suggest that the addition of HIT-IT to a CPI might generate responses in CPI-refractory tumours, thereby overcoming resistance and addressing a current unmet need in unresectable and metastatic melanoma for treatment options following progression after CPI treatment.

## Background

The standard of care for patients with melanoma whose tumour burden is limited and disease spread is confined comprises surgical resection with the intention to cure. In approximately two-thirds of all cases of primary cutaneous melanoma, disease spread begins with locoregional metastasis, with about 50% of patients developing the first metastasis in regional lymph nodes.^1^ Satellite or in-transit metastases also frequently occur at readily accessible cutaneous or subcutaneous locations.^1–4^ Although resection still, theoretically, remains an option in such cases, repeated surgery for locoregional disease might not be the best approach for patients with disease affecting a large anatomic area, for those in whom relapse occurs rapidly following repeated surgery, or for those in whom the morbidity of surgery might outweigh the benefits.^5^ In these, and other, patients with unresectable melanoma, the approval of targeted therapies (such as inhibitors of BRAF and mitogen-activated protein kinase [MEK]) and immune checkpoint inhibitors (CPIs; such as anti-programmed death receptor 1 [PD-1] and anti-cytotoxic T-lymphocyte-associated protein 4 [CTLA-4] agents) has revolutionised outcomes—with CPI treatment, around one-third of patients survive at least 5 years.^6–10^ However, the treatments can result in severe and long-lasting toxicities, and primary or acquired resistance are common.^8–12^ Furthermore, because most Phase 3 trials assessing targeted therapies or CPIs have predominately enrolled patients with stage IV disease, limited efficacy data are available for these agents in the treatment of unresectable stage IIIB–C locoregional melanoma.^13–17^

The need for additional treatment options for unresectable locoregional disease coupled with the accessibility of this type of metastasis has led to increased interest in immunostimulatory agents that can be injected directly into the tumour.^5^ These intratumoural immunotherapies can cause cell lysis, either directly or indirectly, and promote the induction of a local immune response, and might also be associated with the generation of a systemic immune response.^18,19^ Importantly, direct injection of a therapeutic agent into the tumour maximises its concentration at the disease site, promoting a tumour-specific immune response while reducing systemic exposure.^2^ There are anticipated benefits of combining intratumoural therapy with systemic immunotherapies such as CPIs. Not only do they offer different modes of action, but they also have non-overlapping toxicity profiles.^20^

Here, we review the latest data on the development of human intratumoural immunotherapy (HIT-IT), as a single-agent strategy and in combination with CPIs, for unresectable melanoma, alongside practical aspects involved in administering and assessing the response to this therapeutic approach. Although some chemical and local physical and radiation strategies might be defined as intratumoural therapies (e.g. PV-10 [a 10% solution of Rose Bengal], electrochemotherapy, cryotherapy, high-intensity focused ultrasound, irradiation and liposomal and nano-delivery systems) these therapeutic options are not immunotherapies and are thus considered to be outside the scope of this review.^21^

## Ideal characteristics for an intratumoural immunotherapy

An ideal HIT-IT should elicit a measurable biological effect, reflective of the mechanism of action, that should lead to an objective response in the injected tumour.^21^ The ability to generate a local complete response and a durable response (i.e. lasting several months) is important to enable its use as a monotherapy, since it demonstrates the agent’s effectiveness when used on its own.^21–23^ Likewise, local disease control should translate into clinical benefit (e.g. symptom control, delayed disease progression, improved survival).^22^ Ideally, HIT-IT should stimulate a systemic immune response leading to regression of uninjected tumours at locoregional and distant metastatic sites.^18,19,21^ The initiation of a systemic immune response can occur through a variety of mechanisms, including the enhanced release and presentation of tumour antigens, immune cell trafficking and activation and inhibition of immunosuppressive pathways.^24–27^ It is anticipated that HIT-ITs will reach high concentrations in injected lesions, increasing the local bioavailability.^21,28^ Furthermore, by minimising systemic exposure, HIT-IT should be associated with low toxicity compared with systemic immunotherapies.^22,25,29,30^ Moreover, HIT-ITs might be able to reverse resistance to systemic immunotherapies.^31–33^ These attributes indicate that HIT-ITs could be used both as monotherapies and as part of a combination strategy,^28^ and data from the past 5 years suggest synergy between intratumoural and systemic immunotherapies (as described in more detail below).^19,20,34–39^

## Currently available HIT-ITs for unresectable and metastatic melanoma

Talimogene laherparepvec (T-VEC) is the only treatment with regulatory approval for intratumoural administration in unresectable metastatic melanoma, and is recommended in current clinical practice guidelines.^40–44^ Interleukin-2 (IL-2) is included in guidelines as a systemic treatment for unresectable metastatic melanoma,^43,44^ and intratumoural administration has also been investigated.^45–47^

### T-VEC

T-VEC is a genetically modified oncolytic virus that expresses granulocyte-macrophage colony-stimulating factor (GM-CSF).^48,49^ It selectively infects and replicates in tumour cells, which not only leads to cell lysis but also to the release of GM-CSF. This recruits dendritic cells, which, in turn, process and present tumour antigens to cytotoxic T lymphocytes (CTLs), thereby inducing a systemic tumour-specific immune response.^48,49^ T-VEC is approved in Europe for the treatment of adults with stage IIIB, IIIC and IVM1a unresectable melanoma with no bone, brain, lung or other visceral disease, and in the USA for the local treatment of unresectable cutaneous, subcutaneous and nodal lesions in patients with melanoma recurrent after initial surgery.

The approval of T-VEC was based on data from the OPTiM Phase 3 trial (*n* = 436), which demonstrated that the durable response rate (objective response lasting ≥6 months) and overall response rate were significantly higher following treatment with intralesional T-VEC than with subcutaneous GM-CSF in patients with unresectable and/or metastatic stage IIIB–IV melanoma (Table 1).^22^ Efficacy was highest in patients with stage IIIB–IVM1a disease; in this group, T-VEC led to a survival benefit^22^ and demonstrated a tolerable safety profile.^22^ A systemic immune response is suggested by observed reductions in the size of uninjected lesions associated with T-VEC treatment (≥50% reduction in 15–34% of uninjected lesions).^18,22^ This implication is also supported by a prospective Phase 2 trial in which T-VEC led to a significant increase in the number of CD8^+^ T cells, effector and memory cytotoxic lymphocytes (CTLs), natural killer cells, and CTLs expressing PD-1 and CTLA-4 (indicative of immune activation) in uninjected lesions.^50^

**Table 1:** Efficacy and safety of HIT-IT in unresectable stage IIIB–IVM1a melanoma^a^.

Type of intratumoural agent	Study phase and number of patients	Disease stage	Comparator	Primary endpoint	Overall survival (mo)	Overall response (patient level)			Lesion-level responses			DRR	Grade 3/4 AEs
						ORR	CR	PR	Injected lesions	Uninjected lesions	Visceral lesions		
Oncolytic viruses	T-VEC												
	OPTiM Phase 3^18,22,119^ *n* = 436	IIIB–IV (FAS)	GM-CSF	DRR^b^ 16 versus 2%	23 versus 19; HR, 0.79; *p* = 0.051	26 versus 6%; *p* < 0.001	11 versus <1%	16 versus 5%	64%^b^	34%^c,d^	15%^c^	16% versus 2%; OR, 8.9; *p* < 0.001	Incidence of treatment-related grade 3/4 AEs: 11% versus 5%. Grade ≥3 AEs occurred in 36% versus 21% (*p* = 0.003). The only grade 3/4 AE occurring in ≥2% of patients was cellulitis (2% versus <1%)
		IIIB/C-IVM1a (subgroup analysis)			41 versus 25; HR, 0.57; *p* < 0.001	41 versus 2%; *p* < 0.0001	17 versus 0%	24 versus 2%	–	–	–	25 versus 1% *p* < 0.0001	
		IIIB/C (*n* = 131)			HR, 0.48	52 versus 2%	–	–	–	–	–	33 versus 0%	
		IVM1a (*n* = 118)			HR, 0.67	27 versus 2%	–	–	–	–	–	16 versus 2%	
		IVM1b (*n* = 90)			HR, 1.06	6 versus 8%	–	–	–	–	–	3 versus 4%	
		IVM1c (*n* = 96)			HR, 1.08	12 versus 14%	–	–	–	–	–	8 versus 3%	
	Coxsackievirus A21												
	CALM Phase 2^56^ *n* = 57	IIIC–IVM1c	None	irPFS at 6 mo 38.6%	NR	28%	–	–	–	–	–	19%	No grade 3/4 AEs related to study treatment
Cytokines	IL-2												
	Phase 2^30^ *n* = 51	IIIB–IVM1c (FAS)	None	CR and PR at 4 wk	–	–	–	–	79%	–	0%	–	No grade 3/4 AEs recorded
		IIIB/C	–	–	–	–	–	–	97%	–	–	–	
		IV	–	–	–	–	–	–	55%	–	–	–	
	L19–IL-2												
	Phase 2^76^ *n* = 25	IIIB/C	None	CR rate at day 85	–	50%	25%	25%	54%	45%	–	–	A few cases of grade 3 AEs reported: injection-site reaction (rate unknown), injection pain (1 case), transient fatigue (1 case). No grade 4 AEs were reported
	Daromun												
	Phase 2^29^ *n* = 22	IIIC–IVM1a	None	CR in all treated lesions at 12 wk	–	–	–	–	55%	54%	–	–	The only treatment-related grade 3 AE was injection-site reaction (rate unclear)
	Tavokinogene telseplasmid												
	Phase 2^79^ *n* = 51	IIIB–IVM1c	Comparison of 2 cycles (3-mo versus 6-wk)	–	–	35 versus 25%	19 versus 0%	15 versus 25%	–	–	–	–	Grade of AEs not reported. Serious TEAEs were reported in five patients (10%): one case each (2%) of cellulitis, rhabdomyolysis, CVA, dizziness and pulmonary embolism

Full details of lesions eligible for injection not provided for all studies; however, OPTiM (talimogene laherparepvec) and CALM (Coxsackievirus A21) confirmed inclusion of nodal lesions.

*AE* adverse event, *CALM* Coxsackievirus A21 in Late stage Melanoma, *CR* complete response, *CVA* cerebrovascular accident, *DRR* durable response rate, *FAS* full analysis set, *GM-CSF* granulocyte-macrophage colony-stimulating factor, *HIT-IT* human intratumoural immunotherapy, *HR* hazard ratio, *IL-2* interleukin-2, *irPFS* immune-related progression-free survival, *NR* not reached, *OPTiM* Oncovex (GM-CSF) Pivotal Trial in Melanoma, *OR* odds ratio, *ORR* overall response rate, *PR* partial response, *T-VEC* talimogene laherparepvec, *TEAE* treatment-emergent adverse event.

^a^Agents included in this table are those for which monotherapy Phase 2 or 3 clinical trial data are available.

^b^Objective response lasting continuously for ≥6 months.

^c^Reduction in lesion size by ≥50%.

^d^Uninjected non-visceral lesions.

### IL-2

IL-2 is a proinflammatory cytokine that can activate CD8^+^ T cells, regulatory T cells, B cells, macrophages and natural killer cells.^45,51^ Systemically administered IL-2 is approved for the treatment of metastatic melanoma in the USA,^52^ but not in Europe.^53^ To date, intratumoural treatment with IL-2 has only been studied in single-arm trials involving a limited number of patients.^45–47^ Although responses with intratumoural IL-2 appear to be durable (lasting ≥ 6 months), they are largely limited to injected lesions, which suggests that intratumoural IL-2 does not elicit a strong systemic effect—at least, not at the doses and regimens that have been studied.^30^ Intratumoural IL-2 is generally well tolerated.

## Novel agents in development for unresectable and metastatic melanoma

**Fig. 1:**
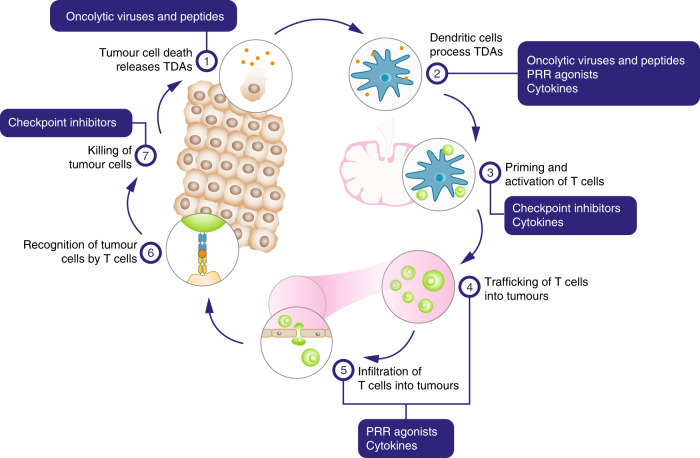
HIT-ITs and the cancer-immunity cycle. The effect of different types of human intratumoural immunotherapy (HIT-IT) agents on different stages of the cancer-immunity cycle. First, oncogenesis causes tumour-derived antigens (TDAs) to be released (step 1). Dendritic cells process the TDAs and present them to T cells on major histocompatibility complex class 1 (MHC1) and class 2 (MHC2) molecules (step 2). The T-cells are primed and activated against the TDAs (step 3), trafficked to the tumour (step 4) and then infiltrated into the tumour bed (step 5). Here the T cells recognise tumour cells through the interaction of the T-cell receptor with the relevant tumour cell antigen bound to MHC1 (step 6). The T cells then kill the tumour cells (step 7), which releases further TDAs to continue the cycle again, with an expanded response. In cancer the cycle does not work optimally; HIT-ITs aim to enhance the cycle at several points in the process. Oncolytic viruses and peptides (e.g. talimogene laherparepvec, Coxsackievirus A21, canerpaturev, RP1, RP2, ONCOS102 and JX-594) act at step 1, causing cell lysis, and in step 2, by causing release of cytokines that recruit dendritic cells to process TDAs. Pattern-recognition receptor agonists (PRRs, e.g. Toll-like receptor-9 agonists SD-101, IMO-2125 and CMP-001; the RIG-I agonist MK4621 and stimulator of interferon genes (STING) agonists ADU-S100 and MK-1454) can act at step 2 by provoking upregulation of cytokines in response to recognition of pathogen-associated molecular patterns (PAMPs) and damage-associated molecular patterns (DAMPs). They can also be involved at steps 4 and 5 by activating TLR-9 signalling to promote T-cell migration and infiltration into tumours. Checkpoint inhibitors (e.g. the anti-CTLA-4 agent ipilimumab and the CD40 agonist APX005M) remove inhibitory signals of T-cell activation, enabling T cell priming and activation at step 3, and modulate active immune response in the tumour bed at step 7. Cytokines (e.g. granulocyte-macrophage colony-stimulating factor, interleukin-2 and daromun) have roles in cancer antigen presentation at step 2, as well as T cell priming, activation and trafficking at steps 3 and 4. Figure adapted from *Immunity* volume 39, Chen, D.S. & Mellman, I. Oncology meets immunology: the cancer-immunity cycle, pages 1–10, Copyright (2013), ref. ^122^ with permission from Elsevier. TDA tumour-derived antigen.

Many agents are being developed for intratumoural use, including other oncolytic viruses and peptides, pattern-recognition receptor (PRR) agonists, immune CPIs and cytokines (see Table 1 and below for further details).^21^ Figure 1 shows how these agents might interact with the cancer-immunity cycle, the process by which cancer cells are effectively killed by an immune response.

### Other oncolytic viruses

Given that the only currently approved HIT-IT is an oncolytic virus, it is not surprising that other oncolytic viruses are undergoing development for the treatment of unresectable and metastatic melanoma.^54^ Coxsackievirus A21 (CVA21) is an enterovirus that preferentially infects tumour cells, leading to cell lysis, which appears to provoke a systemic antitumour immune response even in the absence of the virus encoding an immune component such as GM-CSF or IL-2.^55^ In a single-arm Phase 2 trial, CVA21 led to durable responses lasting 6 months or more.^56^ No Phase 3 trials are currently planned for CVA21 monotherapy in melanoma since the future of these agents is most likely in combination, particularly with immunotherapies. In this regard, trials are ongoing with CVA21 in combination with other therapies (see below). Canerpaturev (formerly HF-10) is a spontaneously occurring, replication-competent mutant strain of herpes simplex virus type 1 that causes lysis of infected cells.^34,57^ Similar to CVA21, canerpaturev is being assessed in combination therapy; no monotherapy trials are ongoing. Other oncolytic viruses are in early clinical development as monotherapy and/or in combination therapy: RP1 and RP2, engineered strains of the herpes simplex virus; ONCOS102, an adenovirus engineered to express GM-CSF; JX-594 (Pexa-Vec), a GM-CSF-expressing poxvirus; and CF33-hNIS, a chimeric poxvirus encoding a human sodium iodide symporter (hNIS).^58–61^

### PRR agonists

Another class of HIT-IT in development comprises the PRR agonists, which include Toll-like receptor (TLR) agonists, stimulator of interferon genes (STING) agonists and retinoic acid-inducible gene I (RIG-I)-like receptor agonists.^21,62^ PRRs are costimulatory molecules that recognise pathogen-associated molecular patterns (PAMPs), such as lipopolysaccharide and other bacterial and viral components, as well as damage-associated molecular patterns (DAMPs) resulting from cellular stress, apoptosis and necrosis.^62^ Recognition of PAMPs/DAMPs leads to upregulation of the transcription of genes involved in inflammatory responses, which encode proinflammatory cytokines, type-I interferons, chemokines and, antimicrobial proteins.^63^ Activation of TLR-9 signalling in plasmacytoid dendritic cells induces production of interferon-α and tumour necrosis factor (TNF)-α, which promotes leucocyte migration and induces synthesis of antimicrobial peptides and cytokines, and promotes phagocytosis in macrophages.^64^ Three TLR-9 agonists (SD-101, IMO-2125 and CMP-001) are in clinical development in combination therapy (see below).^31,32,65,66^ RIG-I-like receptors are cytosolic PRRs that detect viral and endogenous RNA, triggering binding to the mitochondrial antiviral signalling protein (MAVS) and resulting in type-I interferon production.^62^ MK4621 (formerly RGT-100), a synthetic RNA agonist of the RIG-I pathway, has been shown to have antitumour activity in mouse models.^67^ STING is an endoplasmic reticulum transmembrane protein involved in recognition of cytosolic DNA. In tumours, STING pathway activation leads to interferon-β production and T-cell response.^62^ Cyclic dinucleotides have been found to act as immune adjuvants by activating STING, in turn stimulating a proinflammatory immune response;^68^ Phase 1 trials of two intratumoural STING agonists, ADU-S100 and MK-1454, are ongoing.^69–71^

### CPIs

Immune CPIs, such as TNF receptor superfamily agonists (e.g. CD40) and immunoglobulin superfamily antagonists (e.g. PD-1 and CTLA-4), are also in development as intratumoural agents.^21^ The activation of CD40 on antigen-presenting cells initiates their maturation and ability to activate CD8^+^ T cells. Modulation of this pathway in melanoma is being evaluated in a Phase 1/2 trial of an intratumoural CD40 agonist, APX005M.^72^ The feasibility of utilising approved anti-PD-1 systemic therapies as intratumoural agents was demonstrated in a 2018 pilot study; further studies are needed to determine the efficacy of this approach.^73^ Similarly, intratumoural administration of the anti-CTLA-4 antibody ipilimumab plus IL-2 demonstrated the induction of both local and systemic immune responses in a Phase 1 trial; no dose-limiting toxicities were reported.^27^ A Phase 1/2 trial assessing intratumoural ipilimumab plus systemic nivolumab, an anti-PD-1 antibody, is currently recruiting patients.^74^

### Cytokines

Cytokines (e.g. IL-2 and GM-CSF) were among the first HIT-ITs to be assessed in melanoma.^5^ Subsequently, cytokine fusion proteins and plasmids expressing cytokines have been developed with the aim of increasing efficacy.^75,76^ For example, the immunocytokine fusion protein L19–IL-2 (Darleukin) is a targeted form of IL-2 that recognises the extra domain B of fibronectin, which is expressed in cancer-associated blood vessels and extracellular matrix but absent from almost all healthy tissue.^75^ In a single-arm Phase 2 trial, L19–IL-2 resulted in local responses, including local complete responses.^75^ Daromun, another investigational treatment, combines L19–IL-2 and L19–TNF. In a single-arm Phase 2 trial, Daromun led to objective responses in both injected and uninjected lesions. A pivotal trial of Daromun is being conducted in the neoadjuvant setting.^77^

Tavokinogene telseplasmid (also called pIL-12) is a synthetic plasmid encoding the cytokine IL-12, which, when delivered to melanomas in a highly localised manner, leads to a proinflammatory response, resulting in T-cell recruitment and activation. In a Phase 2 trial, tavokinogene telseplasmid demonstrated induction of an antitumour immune response and a high disease control rate in melanoma.^78^ Tavokinogene telseplasmid was given orphan drug status by the US Food and Drug Administration (FDA) in 2017 for the treatment of unresectable metastatic melanoma.^76^

### Other promising HIT-ITs

Several other novel HIT-ITs have shown promising preclinical antitumour activity and are entering clinical trials. AGI-134 is a glycolipid that recruits pre-existing endogenous anti-Gal antibodies to the injected lesion, leading to complement activation and enhanced tumour antigen processing, whereas IMM60 is a non-glycolipid that activates invariant natural killer cells, leading to an antitumour immune response. A Phase 1/2 trial of AGI-134 is currently recruiting patients, and trials of IMM60 are anticipated.^79–81^ IFx-Hu2.0 is a plasmid DNA encoding the streptococcal membrane protein, Emm55, and the first in human Phase 1 study of intratumoural use in melanoma is in progress.^82^ Preliminary laboratory data from the first three patients in the trial suggest the treatment may be associated with decreases in tumour cells and formation of an immune response.^83^ mRNA-2416 is a novel lipid nanoparticle therapeutic agent encoding the TNF receptor ligand OX40L. Results so far delivered from an ongoing Phase 1/2 study indicate that intratumoural mRNA-2416 monotherapy is well tolerated and elevates PD-L1 levels and proinflammatory activity.^84^

### Going forward

Although many HIT-ITs have been investigated, most data at present originate from Phase 2 trials only. Such data are difficult to interpret, because early-stage efficacy might not reflect the results of Phase 3 trials:^85–87^ for example, despite promising efficacy in Phase 2 trials, the plasmid-DNA-based intratumoural immunotherapy velimogene aliplasmid (a plasmid–lipid complex comprising the DNA sequences encoding HLA-B7 and β2 microglobulin) either provided no benefit or reduced survival compared with standard of care in Phase 3 trials.^86^ This result highlights the need for caution when interpreting data from single-arm, non-randomised trials in small and highly selected patient populations, as well as emphasising the importance of mechanistic tumour biology research to enable rational drug and clinical trial design.

## Combining intratumoural and systemic immunotherapies

As noted, many intratumoural agents in development are being assessed in combination with systemic CPIs, reflecting the likely future clinical application of intratumoural therapy.

### HIT-ITs might enhance the response to CPIs...

Although CPIs provide an effective therapeutic approach as monotherapy in melanoma,^6,7^ only a subset of patients initially respond, and a substantial proportion of responders subsequently develop resistance and relapse.^88^ Data suggest that therapeutic responses to immunotherapy can, to some extent, be predicted by the presence of tumour immune cell infiltration. Three tumour immune profiles correlate with response to CPIs: inflamed or ‘hot’ tumours exhibiting immune cell infiltration are likely to respond, whereas ‘cold’ immune-excluded tumours with immune cells surrounding, but not infiltrating, the tumour, and immune desert tumours, characterised by a complete lack of immune cells, are less likely to respond. HIT-ITs that elicit a local immune response have been shown to promote immune cell infiltration into the tumour in both injected and uninjected lesions.^26,37,50^ Consequently, by altering the tumour microenvironment and converting a non-responsive ‘cold’ tumour into a responsive ‘hot’ tumour, HIT-IT might enhance response to systemic immunotherapies.^26,37,38^

### …and CPIs might enhance the response to HIT-ITs

On the other hand, CPIs might also enhance the response to HIT-IT. Cancer cells can activate immune checkpoint pathways to downregulate the response to local immunostimulation, thus limiting the response to intratumoural monotherapy.^89,90^ Indeed, a 2018 Phase 2 trial of T-VEC showed that this therapy leads to an increase in the number of CTLs expressing PD-1 and CTLA-4.^50^ Blocking PD-1 or CTLA-4 might restore suppressed antitumour immune responses and enhance the ability of T cells (which have been primed by intratumoural injection) to recognise and kill tumour cells.^35,91^ CPIs might also enhance the systemic effects of some HIT-ITs.^35,37,38,50^ Therefore, combination therapy might result in improved clinical activity beyond what would be expected with either agent alone. Additionally, owing to the low toxicity of HIT-IT, combination therapy has the potential to be tolerated at effective doses.

### Combination trials of HIT-ITs with pembrolizumab or ipilimumab

Given the therapeutic promise of combining intratumoural and systemic immunotherapies, several combination trials have been conducted or are ongoing (Table 2). Data indicating the synergistic activity of such combinations are available from the Phase 1b/3 MASTERKEY-265/KEYNOTE-034 trial of T-VEC plus the anti-PD-1 agent pembrolizumab versus pembrolizumab alone. In the combination arm, two injections of T-VEC were given before pembrolizumab was initiated. Although single-agent T-VEC increased CD8^+^ T-cell infiltration into the tumour, in both injected and uninjected lesions, and increased the numbers of systemic circulating CD4^+^ and CD8^+^ T cells, combination therapy was associated with greater clinical benefit than that seen previously with either agent alone, with no additional toxicity.^37,92^ Longer-term follow-up (median 36.8 months) of the Phase 1b part of the trial suggests that this combination induces a high rate of complete responses (43%), with a 3-year survival rate of ~70%.^93^ The same combination is currently being evaluated in the large randomised Phase 3 MASTERKEY-265/KEYNOTE-034 trial.^94^

**Table 2:** Efficacy of combination therapy with HIT-IT and systemic immunotherapies.

Type of intratumoural agent	Agent and study phase	Enrolled patients	Disease stage	Comparator	Primary endpoint	Anti–PD-1 refractory/previous treatment	ORR	CR
*Combination trials with systemic ipilimumab*								
Oncolytic viruses	T-VEC Phase 2^39,99^	198	IIIB–IVM1c	Systemic ipilimumab	ORR (in accordance with irRC)	2% versus 3% had previously received anti–PD-1 therapy	39% versus 18%; OR, 2.9; *p* = 0.002 Stage IIIB–IVM1a: 44% versus 19%; OR, 3.3; *p* = 0.007 Stage IVM1b/c: 33% versus 16%; OR, 2.6; *p* = 0.09	13% versus 7%
	Coxsackievirus A21 MITCI Phase 1b^34,120^	26	IIIC–IVM1c	None	Safety	–	57% (23% in 15 patients with previous anti–PD-1 treatment)	–
	Canerpaturev Phase 2^58,112^	46	IIIB–IV	None	BORR at 24 wk	–	41% Stage IIIB–IVM1a: 47% Stage IVM1b/c: 20%	18%
	Canerpaturev Phase 2^101,121^	28	IIIB–IVM1c	None	BORR at 24 wk	89% of patients had previously received anti–PD-1 therapy	7%	0%
PRR agonists	IMO-2125 ILLUMINATE-204 Phase 1/2^31^	21	III–IV	None	To determine RP2D	All patients had previously received anti–PD-1 therapy	38%	10%
Cytokines	IL-2 Phase 2^2^	15	Pretreated melanoma with distant metastasis	None	DCR at wk 12	–	0%	–
*Combination trials with systemic pembrolizumab*								
Oncolytic viruses	T-VEC MASTERKEY-265 Phase 1b/3 (data from Phase 1b)^37,93,94^	21	IIIB–IVM1c	Systemic pembrolizumab (for Phase 3 part only)	Incidence of DLTs	–	62%	33%
	Coxsackievirus A21 CAPRA Phase 1b^36^	50 (19 included in safety analysis)	IIIB/C–IV	None	Safety	–	60%	–
PRR agonists	SD-101 SYNERGY-001/KEYNOTE-184 Phase 1b/2^66,67^	87	IIIB–IVM1c	None (SD-101 given at 2 or 8 mg per lesion)	Safety, evaluate the expression of IFN-inducible genes in whole blood 24 h after SD-101 administration as a pharmacodynamic marker of SD-101 activity, determine the RP2D	All patients were naïve to anti–PD-1/L1 therapy	70% (SD-101 2 mg/lesion) 48% (SD-101 8 mg/lesion)	11% (SD-101 2 mg/lesion) 5% (SD-101 8 mg/lesion)
	SD-101 SYNERGY-001/KEYNOTE-184 Phase 1b/2^33^	30	IIIC–IV	None	ORR	All patients were resistant or refractory to anti–PD-1 therapy	21%	3%
	CMP-001 Phase 1b^32^	69	III–IVM1d	None	To determine RP2D	All patients had previously received anti–PD-1 therapy. In total, 91% of patients had progressive disease and 9% had stable disease on previous anti–PD-1 therapy	22%	–
Cytokines	Tavokinogene telseplasmid (pIL‐12) Phase 2^79,96^	23	IIIB–IVM1c	Plasmid IL-12 monotherapy	–	–	50 versus 25–35%	41 versus 0–19%

Full details of lesions eligible for injection not provided for all studies; however, Phase 2 study of talimogene laherparepvec with systemic ipilimumab confirmed inclusion of nodal lesions.^39^

*BORR* best overall response rate, *CAPRA* CAvatak and PembRolizumab in Advanced melanoma, *CR* complete response, *DCR* disease control rate, *DLT* dose-limiting toxicity, *HIT-IT* human intratumoural immunotherapy, *IFN* interferon, *IL-12* interleukin-12, *IL-2* interleukin-2, *irRC* immune-related response criteria, *MITCI* Melanoma Intra-Tumoral Cavatak and Ipilimumab, *OR* odds ratio, *ORR* overall response rate, *PD-1/L1* programmed death receptor 1/programmed death receptor ligand 1, *PRR* pattern recognition receptor, *RP2D* recommended phase 2 dose, *T-VEC* talimogene laherparepvec.

Systemic pembrolizumab is being assessed in combination with various other HIT-ITs, with promising early results. Interim data from a Phase 1b/2 trial combining the intratumoural TLR-9 agonist SD-101 with pembrolizumab demonstrated that the combination is well tolerated and leads to increased tumour immune cell infiltration, as well as inducing tumour shrinkage, in injected and uninjected lesions—including those in distant metastases.^65,66^ Pembrolizumab is also being investigated in a Phase 2 trial with intratumoural tavokinogene telseplasmid^78,95^ and in a Phase 1b trial with the oncolytic virus CVA21. A Phase 2 trial is also planned to investigate pembrolizumab with CVA21.^96^ Apart from T-VEC plus pembrolizumab, none of these combinations is currently being assessed in Phase 3 trials.

T-VEC has also been assessed in a randomised Phase 1b/2 trial in combination with ipilimumab versus ipilimumab alone;^97^ the results suggest that this combination is tolerable and might have greater efficacy than either agent alone.^39,98,99^ Other ipilimumab and intratumoural combinations have shown efficacy in Phase 1b or 2 trials, including with the oncolytic viruses CVA21 and canerpaturev.^34,100^ However, a Phase 2 trial of ipilimumab combined with intratumoural IL-2 reported no objective responses.^2^ Currently there are no ongoing or planned Phase 3 trials of any of these combinations.

### Use of HIT-IT following anti-PD-1 therapy

For patients who have previously received an anti-PD-1 agent, treatment options are limited.^88^ Data from the past 3 years—particularly from studies using TLR-9 agonists—indicate that treatment regimens incorporating a HIT-IT can lead to responses in patients who have previously received, or who have progressed following, anti-PD-1 therapy (Table 2).^31–33,101^ Limited data are available on the efficacy of HIT-IT as monotherapy in patients who have previously received an anti-PD-1 therapy.

Several studies are investigating whether combining a HIT-IT with ipilimumab can provide additional activity. In a Phase 1/2 trial, addition of the TLR-9 agonist IMO-2125 (tilsotolimod) to ipilimumab revived the immune response in injected and uninjected anti-PD-1-resistant tumours.^31^ A randomised Phase 3 trial assessing IMO-2125 plus ipilimumab in patients who have progressed on previous anti-PD-1 therapy is ongoing.^102^ Phase 2 data show that the addition of canerpaturev to ipilimumab can lead to responses in a minority of patients previously treated with anti-PD-1 therapy.^101^

Early phase data indicate that the addition of a HIT-IT to an anti-PD-1 agent might restore response and fundamentally overcome resistance to anti-PD-1 therapy. In a Phase 1b trial, the TLR-9 agonist CMP-001 was able to overcome resistance to PD-1 inhibition when combined with pembrolizumab.^32^ Likewise, in a Phase 1b/2 trial, addition of the TLR-9 agonist SD-101 to pembrolizumab restored tumour sensitivity to PD-1 inhibition in refractory tumours; responses were seen in both injected and uninjected lesions.^33^ A Phase 2 trial of T-VEC plus pembrolizumab is ongoing in patients with advanced melanoma whose disease progressed following anti-PD-1 therapy.^103^

For future analyses on the sequence of HIT-ITs, it will be important to ascertain if the response to these agents differs among patients who progress following initial response to CPI treatment compared with those who never respond to CPI treatment and/or patients who have previously received a CPI and discontinued for other reasons (e.g. poor tolerability). This will help to identify the most appropriate HIT-IT to use in different clinical situations. Another interesting area for future research is the potential to give HIT-IT to patients who experience progression despite adjuvant CPI therapy, as many of these patients progress with accessible locoregional disease only.^104^

### HIT-ITs in combination with targeted therapies

There is also interest in combining HIT-ITs with BRAF and MEK inhibitors such as dabrafenib and trametinib. This approach aims to specifically target the BRAF driver mutation, which is present in around 50% of malignant melanomas.^105^ The combination of MEK inhibition and T-VEC has shown increased melanoma tumour cell death in vitro^106^ and a Phase 1 trial of dabrafenib, trametinib and T-VEC is ongoing.^107^

## Practical implications of HIT-IT for the clinical management of unresectable and metastatic melanoma

### Patient selection

From a practical perspective, HIT-IT can only be administered to patients with lesions that are visible, palpable or detectable by ultrasound or other imaging techniques. Key considerations for the selection of patients for HIT-IT monotherapy are described in Box 1. The decision to select a patient for HIT-IT should result from discussions within a multidisciplinary team. These considerations will change if a HIT-IT is approved in combination with a systemic CPI. Similarly, a better understanding of the chances of obtaining a response will help to weigh the risk:benefit consideration in the case of less accessible lesions (e.g. lung metastases).

Box 1. Patient selection for HIT-IT monotherapy Several key considerations exist for HIT-IT monotherapy patient selection:

When surgery is no longer an option owing to a number of reasons^22,108,123,124^

- tumour location, leading to unreasonable morbidity (e.g. cutaneous head and neck melanoma)
- risk of surgical complications
- presence of numerous in-transit lesions
- disease recurrence despite multiple surgical interventions

As an alternative to systemic therapy in patients

- for whom systemic therapies are contraindicated or poorly tolerated
- with slowly progressing disease or locoregional progression but stable visceral metastases who wish to avoid systemic therapy
- who wish to preserve systemic therapies for later treatment lines in the event of disease progression

### Lesion mapping and injection

To ensure accurate drug delivery, response and evaluation, lesion mapping using clinical evaluation (i.e. palpation and imaging) should be conducted to identify and measure lesions for injection. Lesions that will not be injected should also be mapped to enable the assessment of systemic responses. Ultrasound provides an accessible option for the mapping of most lesions (including subcutaneous and nodal), as well as allowing the measurement of 3D tumour volume, and can be more accurate than palpation for the determination of lesion size. Computed tomography (CT), positron emission tomography (PET-CT) and, in rare cases, magnetic resonance imaging can also be considered for mapping deep lesions or those located in the extremities. Before initiating HIT-IT, it is important to record the tumour load throughout the patient, the tumour volume available for injection and the total volume of agent injected before response evaluation. Both injected and uninjected lesions (including visceral lesions) should be measured before treatment and compared with previous measurements: a lesion tracking sheet and high-resolution photographs could facilitate lesion tracking.

It is beyond the scope of this review to detail handling and administration for HIT-IT; these topics have been covered extensively by Marabelle et al.,^21^ Gutzmer et al.^108^ and Harrington et al.^109^. Briefly, most cutaneous, subcutaneous and superficial lymph node lesions can be injected under ultrasound guidance.^21^ Multiple injections can be administered to a single lesion, and multiple lesions can be injected at the same visit.^21^ The choice of whether to use the same needle for all lesions or individual needles for each lesion might depend on the type of HIT-IT used and the risk of drug exposure to the patient or healthcare professional.^21,41^ Intratumoural injections should be performed by a trained healthcare professional (e.g. a nurse, physician, radiologist, interventional radiologist or surgeon).^21^

Electroporation is sometimes employed for delivery of tavokinogene telseplasmid and other plasmids. After injection of the plasmid into the lesion, application of electroporation pulses locally permeabilise and transduce the cells.^110^ The precision of the technique is an advantage. Only cells that are exposed to both the plasmid and the electrical field will undergo transfection, enabling local, targeted delivery.^110^

### Safety and tolerability of intratumoural injections

As mentioned, most reported adverse events associated with HIT-IT have generally been mild and might not require active treatment. Injection-site reactions are frequent, but they tend to resolve within 24–48 h and can be helped with ice and pain relief. Local anaesthetic can be given ~30 min before the procedure. Careful wound care is important to help to avoid skin infections. In the event of cellulitis, oral or intravenous antibiotics should be administered as required and blood samples taken for culture.^111^ Although HIT-IT is generally associated with low toxicity, adverse events such as fatigue, chills and pyrexia have been reported in clinical trials and it is important to manage the patient’s expectations of tolerability to ensure that they remain on treatment.^18,22,112^

### Assessment of response

Current trials of HIT-ITs have used a range of response criteria. Thorough and consistent assessments of response will be required in future clinical trials to properly evaluate the plethora of HIT-ITs in development. The Response Evaluation Criteria in Solid Tumors (RECIST v1.1) were designed to measure responses to cytotoxic agents, and might not be suitable for evaluating HIT-ITs: according to RECIST v1.1, new lesions define progressive disease, but the appearance of new lesions can precede prolonged disease stabilisation in response to immune checkpoint blockade.^113^ Indeed, pseudoprogression, in which a tumour appears to grow as a result of a treatment effect rather than true disease progression, has been reported to occur in patients receiving HIT-IT;^18^ in such cases, discontinuing treatment at the point of apparent initial disease progression might not be appropriate. The immune-related response criteria (irRC) and immune-related (ir)RECIST partially ameliorate these issues by requiring confirmation of progressive disease by consecutive imaging assessment at least 4 weeks from the date of the first documentation and by incorporating the measurement of new lesions into the sum of lesions.^114–116^ irRECIST further attempts to harmonise data collection and to clarify response measurement that allows for pseudoprogression. New lesions are assessed separately. Importantly, the progressive disease thresholds for irRECIST are aligned with RECIST v1.1, allowing comparisons to be made between trials and historical data.

European Society for Medical Oncology (ESMO) recommendations on the development of HIT-ITs propose that, for clinical trials, RECIST v1.1 is used for the overall assessment of tumour response, iRECIST is used for assessing responses of injected and uninjected tumours, and the duration of response for both injected and uninjected lesions should be reported.^21^ We are in agreement with the ESMO recommendations that injected lesions are evaluated separately from uninjected lesions, to characterise systemic effects in clinical trials.^21^ As a result, the evaluation of HIT-IT in clinical trials is more complex than for conventional therapies, due to the need to differentiate lesions (e.g. ‘target injected’, ‘target non-injected’, ‘non-target’, ‘new target’ and ‘new non-target’). Furthermore, visceral lesions must be considered in the response criteria; responses to HIT-IT have been reported in uninjected visceral lesions, and advances in imaging guidance techniques now allow visceral lesions to be directly injected.^31^ Such complexity in response assessment requires specially trained radiologists.

These assessment criteria are currently likely to be too complicated and not relevant for real-world practice, in which treatment decisions are based on patient-level responses, and harmonisation on how to evaluate tumour response to HIT-IT is still awaited. As HIT-IT becomes more widely used, the link between formal response evaluation, clinical benefit and decision-making in practice will evolve further.

## Conclusions

The efficacy and tolerability of HIT-IT for the treatment of melanoma provide proof-of-concept for this modality. Early data indicate the existence of a synergy between HIT-IT and CPIs, and it is likely that future clinical use will focus on the combined use of these agents; indeed strategies that combine treatments that have different modes of action without overlapping toxicities are likely to feature in future research. Furthermore, there is a high unmet need in unresectable and metastatic melanoma for treatment options following progression after PD-1 inhibition, and data indicate that adding HIT-IT to systemic agents can lead to responses in anti-PD-1-refractory tumours, thereby overcoming resistance.

The development of HIT-IT has focused on unresectable disease; however, there is now interest in the efficacy of this approach in the neoadjuvant setting, and early data suggest that neoadjuvant HIT-IT could prevent recurrence following surgery in patients with resectable melanoma.^117,118^ Additionally, although it has been widely assessed in melanoma, HIT-IT could be considered for use in any tumour that is injectable (including under image guidance).^21^ Consequently, a plethora of HIT-ITs are in early-stage clinical development for the treatment of a range of solid tumour types and lymphoma.

## Data Availability

Not applicable (no datasets were generated or analysed during article).
